# The role of ZNF143 overexpression in rat liver cell proliferation

**DOI:** 10.1186/s12864-022-08714-2

**Published:** 2022-07-02

**Authors:** Bingyu Ye, Wenlong Shen, Chunyan Zhang, Mengli Yu, Xinru Ding, Man Yin, Yahao Wang, Xinjie Guo, Ge Bai, Kailin Lin, Shu Shi, Ping Li, Yan Zhang, Guoying Yu, Zhihu Zhao

**Affiliations:** 1 State Key Laboratory of Cell Differentiation and Regulation, College of Life Sciences, Henan Normal University, Xinxiang, 453007 China; 2 Fengtai District, Beijing Institute of Biotechnology, No. 20, Dongdajie Street, Beijing, 100071 China; 3 Institute of Genetics and Developmental Biology, Chinese Academy of Sciences, Beijing, 100101 China

**Keywords:** ZNF143, Binding site, Cell proliferation, CUT&Tag

## Abstract

**Background:**

Zinc finger protein 143(ZNF143), a member of the Krüppel C2H2-type zinc finger protein family, is strongly associated with cell cycle regulation and cancer development. A recent study suggested that ZNF143 plays as a transcriptional activator that promotes hepatocellular cancer (HCC) cell proliferation and cell cycle transition. However, the exact biological role of ZNF143 in liver regeneration and normal liver cell proliferation has not yet been investigated.

**Methods:**

In our study, we constructed a stable rat liver cell line (BRL-3A) overexpressing ZNF143 and then integrated RNA-seq and Cleavage Under Targets and Tagmentation (CUT&Tag) data to identify the mechanism underlying differential gene expression.

**Results:**

Our results show that ZNF143 expression is upregulated during the proliferation phase of liver regeneration after 2/3 partial hepatectomy (PH). The cell counting kit-8 (CCK-8) assay, EdU staining and RNA-seq data analyses revealed that ZNF143 overexpression (OE) significantly inhibited BRL-3A cell proliferation and cell cycle progression. We then performed CUT&Tag assays and found that approximately 10% of ZNF143-binding sites (BSs) were significantly changed genome-wide by ZNF143 OE. However, CCCTC-binding factor (CTCF) binding to chromatin was not affected. Interestingly, the integration analysis of RNA-seq and CUT&Tag data showed that some of genes affected by ZNF143 differential BSs are in the center of each gene regulation module. Gene ontology (GO) enrichment and Kyoto Encyclopedia of Genes and Genomes (KEGG) pathway analyses indicated that these genes are critical in the maintenance of cell identity.

**Conclusion:**

These results indicated that the expression level of ZNF143 in the liver is important for the maintenance of cell identity. ZNF143 plays different roles in HCC and normal liver cells and may be considered as a potential therapeutic target in liver disease.

**Supplementary Information:**

The online version contains supplementary material available at 10.1186/s12864-022-08714-2.

## Background

Liver diseases affect the health of people worldwide. In particular, nonalcoholic fatty liver disease (NAFLD) and alcohol-related liver disease (ALD) have emerged as common forms of liver disease [1]. Many cases progress to end-stage liver diseases, such as liver failure, cirrhosis, and hepatocellular cancer (HCC). Therefore, it is particularly important to identify effective treatment strategies for liver diseases.

The liver performs a wide variety of physiological functions including metabolism, lipid synthesis, nutrient storage, and detoxification. These functions are essential for maintaining homeostasis and are mostly performed by parenchymal hepatocytes [2]. Moreover, the liver has a unique regenerative capacity after mild injury, resection, or viral infections [3]. During liver regeneration, hepatocytes are the primary effector cells for proliferation and help restore lost liver mass [4, 5]. Thus, understanding the mechanisms regulating liver regeneration is important for developing better therapeutic agents to treat liver diseases. Currently, it is generally understood that liver regeneration is regulated by several key signaling pathways, including the IL-6/Jak/STAT3, PI3-K/PDK1/Akt, Hedgehog, WNT, and β-catenin, Hippo, and the Yap pathway [6, 7]. However, the cellular and molecular mechanisms that regulate liver regeneration are poorly understood.

As a ubiquitously expressed eukaryotic transcription factor (TF), ZNF143 was first discovered in *Xenopus laevis,* and it is a sequence-specific transcriptional activator of both RNA polymerase II (RNA pol II) and RNA polymerase III (RNA pol III) [8, 9]. Studies have revealed that ZNF143 is involved in various cellular and biological processes, including cell growth, cell proliferation, cell cycle regulation, cancer development, DNA repair, embryonic development, hematopoietic stem and progenitor cell identity, and genetic disorders [10–22]. Recently, an increasing number of studies have shown that ZNF143 is a critical regulator of chromatin loop formation. Genome-wide binding sites (BSs) have also revealed that ZNF143 is participating in CTCF-Cohesin-mediated chromatin interactions [23–26]. Notably, two recent studies focusing on human liver tissue and hepatocytes suggested that ZNF143 is involved in HCC cell proliferation and HBV replication [27, 28]. However, the exact biological role of ZNF143 in liver regeneration and normal liver cell proliferation has not yet been investigated.

In the present study, we found that ZNF143 displayed a dynamic expression pattern during rat liver regeneration after 2/3 PH, and was significantly upregulated during the proliferation phase. To evaluate the role of ZNF143 in liver cell proliferation, we constructed a ZNF143 overexpression (OE) cell model and found that ZNF143 significantly suppressed cell proliferation by inhibiting the cell cycle. Finally, we investigated the mechanism of ZNF143 in cell proliferation using the Cleavage Under Targets and Tagmentation (CUT&Tag) method. Importantly, integrated RNA-seq and CUT&Tag analysis revealed that differential gene expression is correlated with the strength of ZNF143 binding, and the genes that were affected by ZNF143 overexpressed BSs were located in the center of gene expression modules. Our findings shed light on the role of ZNF143 in regulating liver cell proliferation. More importantly, the results provide a foundation for elucidating the function of ZNF143 during liver regeneration.

## Methods

### Animals

Male Sprague Dawley (SD) rats were purchased from Beijing Vital River Laboratory Animal Technology Co., Ltd., Beijing, China. All rats were housed under a light: dark cycles with free access to water and food. All animal experiments were performed with the approval of the ethics committee at the Beijing Institute of Biotechnology, Beijing, China, and conformed to the relevant regulatory standards. All animal studies were completed in the experimental animal center of the Academy of Military Medical Sciences, China (license number: SCXK- (Army) 2007–004, licensed by the Ministry of Science and Technology of China).

#### PH

At 9–10 weeks of age, PH was performed based on Mitchell and Willenbring (2008) [29]. Rats were sacrificed at 0 h, 2 h and 24 h after PH to obtain liver tissue (9 mice, *n* = 3 per time point). According to the requirement of immunohistochemistry (IHC) or western blot to prepare liver samples.

### Cell culture and ZNF143 OE cell line construction

Rat liver cell line BRL-3A was purchased from cell bank of School of Basic Medicine of Peking Union Medical College (Beijing, China) and cultured in DMEM (Life technologies, Waltham, MA) containing 10% fetal bovine serum (FBS; Gibco, Grand Island, NY) with 1% penicillin/streptomycin at 37 °C in 5% CO_2_.

The rat ZNF143 sequence was ligated into the pCDH-CMV-MCS-EF1- copGFP-T2A-puro vector (Shanghai Generay Biotech Co., Ltd). The resulting plasmid was called pCDH-CMV-MCS-EF1-copGFP-T2A-puroZNF143. As a mock control (GFP alone), pCDH-CMV-MCSEF1-copGFP-T2A-puro was used. For recombinant lentivirus packaging, HEK293T cells were transfected with lentiviral vectors and packaging plasmids mix by calcium phosphate transfection. Viruses were collected, filtered through 0.45 μm membrane and utilized to transduce BRL-3A cells. The stable overexpressing cell lines were selected with puromycin.

### CCK-8 assay

ZNF143 OE cells were seeded into 96-well plates at 3 × 10^3^ cells/well and incubated for 24, 48 and 72 h before adding 10 μL of CCK-8 solution per well for 2 h. Absorbance (450 nm) was measured using a Biotek reader (ELx800, Winooski, VT).

### EdU assay

Cells were incubated at 37 °C with 50 μmol/L EdU (RiboBio, Guangzhou, China) for 2 h, after fixing in 4% paraformaldehyde solution at 4 °C for 30 min, cells were treated with 0.5% Triton X-100 at room temperature for 10 min. Finally, the cells were incubated with an Apollo® reaction cocktail for 30 min, For DNA staining, cells were incubated with 5 μg/mL Hoechst 33,342 for 30 min in dark room. Analysis was performed by fluorescence microscopy.

### Total RNA extraction and qRT-PCR

Total RNA was isolated with TRIzol reagent (QIAGEN) and reverse-transcribed into cDNA using GOScript™ Reverse Transcription System (Promega). Quantitative real-time polymerase chain reaction (qRT-PCR) were performed using GoTaq® qPCR Master Mix (Promega) on a LightCycler® 96 Instrument (Roche) according to the manufacturer’s instructions. Rat β-actin was used as an internal reference gene. The primers sequences for genes are as follows: *ZNF143* forward: CAGGTCAAGGTGATGATGTTCTTAAAGGGT and reverse: GGCCTGCATGTCGGCTTGAGATATG; *β-actin* forward: ACATCCGTAAAGACCTCTATGCCAACA and reverse: GTGCTAGGAGCCAGGGCAGTAATCT.

### IHC

Liver tissues were fixed with 4% paraformaldehyde (Sigma-Aldrich) at room temperature (RT) for more than 24 h, and embedded in paraffin. Tissue sections were deparaffinized with xylene and rehydrated with graded series of ethanol (absolute, 95%, 90%, 80%, 75%, respectively, and distilled water), followed by wash twice with PBS-T for 5 min. Antigen retrieval was performed for 10 min in 10 mM sodium citrate buffer (pH 6.0) at 95–100 °C followed by wash with PBS-T for 5 min at RT. Immediately, tissue sections were incubated in 3% hydrogen peroxide for 10 min to block endogenous peroxidase activity. Tissue sections were then washed with PBS-T for 5 min and blocked (Immunostaining Blocking Solution: 2% normal goat serum, 2% bovine serum albumin (BSA) and 0.1% Triton-X in PBS) for 30 min at RT. Tissue sections were then incubated in humidified chamber for over 16 h at 4 °C with ZNF143 primary antibody (Proteintech, 16,618–1-AP) (1:100 in TBST). Tissue sections were washed three times with PBS-T for 5 min and incubated at RT for 1 h with secondary antibody (goat anti rabbit). After wash twice with PBS-T for 5 min at RT, sections were incubated with streptavidin peroxidase (Beyotime) for 10 min at RT and the color was developed using a DAB substrate kit (Beyotime).

### Western blot

Cell lysis buffer for Western and IP kit and Nuclear and Cytoplasmic Protein Extraction Kit were purchased from Beyotime Biotechnology. Protein lysates were mixed with 5 × SDS sample buffer (Beyotime) and heated at 95 °C for 8 min. Next, a total of 30 μg of protein lysates were run on 12% SDS-PAGE. Proteins were then transferred to PVDF membrane. The membrane was blocked with 5% milk in TBST for 1–2 h at RT and incubated with the primary antibodies overnight at 4 °C. The membrane was next incubated with goat anti-rabbit HRP-conjugated secondary antibody for 1 h at RT. Finally, protein bands were performed with ECL Prime reagent and chemiluminescence signals were detected by Odyssey XF (LI-COR Biosciences). Antibodies used in this study were anti-ZNF143 (Proteintech, 16,618–1-AP, 1:1000), anti-β-actin (Cell Signaling Technology, #4967S, 1:2000), anti-Lamin A/C(ThermoFisher, MA1-06,102, 1:1000), anti-GAPDH (Cell Signaling Technology, #8884S, 1:2000).

### Immunofluorescence (IF)

The cells were fixed with 4% paraformaldehyde at RT for 20 min and then permeabilized with 0.1% Triton X-100. After blocking with 1% BSA, cells were incubated with primary antibody (anti-ZNF143, Proteintech, 16,618–1-AP, 1:200).

at 4 °C for more than 16 h. Finally, cells were labeled with fluorescent-labelled secondary antibodies and nuclear stained with DAPI.

### RNA-seq

Total RNA was extracted using the TRIzol reagent (QIAGEN). 3 μg total RNA was used to prepare for each RNA-seq library. The libraries were prepared using Illumina TruSeq Stranded mRNA Library Prep Kit Set A (RS-122–2101; Illumina). Libraries quality and quantity were estimated with TapeStation (Agilent Technologies). Libraries were sequenced on Illumina Novaseq 6000 (150-bp paired ends).

### CUT&Tag

CUT&Tag was performed based on a protocol published by Kaya-Okur et.al [30]. In brief, cells were harvested, counted (500,000 cells) and centrifuged for 3 min at 600 × g at RT. Cells were washed twice in 1.5 mL Wash Buffer (20 mM HEPES pH 7.5; 150 mM NaCl; 0.5 mM Spermidine; 1 × Protease inhibitor cocktail, EDTA free). 10 μL concanavalin A coated magnetic beads (Bangs Laboratories) were added per sample and incubated at RT for 15 min. The supernatant was removed and bead-bound cells were resuspended in 50 μL Dig-wash Buffer (20 mM HEPES pH 7.5; 150 mM NaCl; 0.5 mM Spermidine; 1 × Protease inhibitor cocktail; 0.05% Digitonin) containing 2 mM EDTA. The primary antibody of ZNF143 (Proteintech, 16,618–1-AP) or CTCF (Millipore, 07–729) was diluted 1:50 in 50 μL of Dig-Wash buffer and then incubated on a rotator overnight at 4 °C. The primary antibody was removed and an appropriate secondary antibody (goat anti-rabbit) was diluted 1:100 in 100 μL of Dig-Wash buffer and cells were incubated at RT for 30 min. Cells were washed using 1 mL Dig-Wash buffer to remove unbound antibodies. A 1:200 dilution of pAG-Tn5 (Novoprotein, N259-YH01-01B) was prepared in Dig-300 Buffer (0.05% Digitonin, 20 mM HEPES, pH 7.5, 300 mM NaCl, 0.5 mM Spermidine, 1 × Protease inhibitor cocktail) and incubated at RT for 1 h. Cells were washed twice with 1 mL Dig-300 Buffer to remove unbound pAG-Tn5. Then, cells were resuspended in 50 μL Tagmentation buffer (10 mM MgCl_2_ in Dig-wash Buffer) and incubated at 37 °C for 1 h. Next, 1 μL of 10% SDS was added to 50 μL of sample and incubated at 55 °C for 10 min to stop tagmentation. To extract the DNA, 1.5 × Ampure XP beads (Beckman Counter) were added to each tube. The final DNA products were eluted with 20 μL Millipore water and prepared to amplify libraries. PCR cycling conditions: 72 °C for 5 min; 98 °C for 30 s; 12 cycles of 98 °C for 10 s and 63 °C for 30 s; final extension at 72 °C for 1 min. The final libraries were purified by adding 1.1 × Ampure XP beads and eluted in 30 μL 10 mM Tris pH 8.0. The size distribution of libraries was determined by Agilent 4200 TapeStation analysis. Libraries were sequenced on Illumina Novaseq 6000 (150-bp paired ends).

### Data processing and analysis

*RNA-seq:* RNA-seq reads were aligned against rat rn6 genome assembly using STAR2.5.3a. The mapped reads were counted using HTSeq0.8.0 toolkit. Batch effects were removed by ComBat-seq batch effect removal algorithm (https://github.com/zhangyuqing/ComBat-seq). Differentially expressed genes (DEGs) analysis was performed using the DESeq2 1.18.1 package in R3.4.3, DEGs were called by FDR < 0.05 and fold change > 2 thresholds. GO enrichment analysis was performed using the clusterProfiler package. Significant pathways were identified based on the KEGG [31–33].

*CUT&Tag:* Paired-end reads were aligned to the reference rat genome rn6 using Bowtie2 (version 2.2.9) with options: –local–very-sensitive-local–no-unal–no- mixed–no-discordant–phred33 -I 10 -X 700. PCR duplicates were removed by using Picard MarkDuplicates. Heatmaps were generated by using deepTools. Peaks were called by MACS2 (version 2.1.1.20160309) with default parameters.

### Statistical analysis

Statistical analysis was carried out using GraphPad Prism 6 software (GraphPad Software, San Diego, CA, USA). The results are presented as means ± SD unless otherwise stated. Comparisons of two groups were performed using two-tailed and unpaired Student’s t-tests. Statistical significance is displayed as *p < 0.05 or **p < 0.01 unless specified otherwise.

## Results

### ZNF143 dynamic expression pattern during liver regeneration

**Fig. 1:**
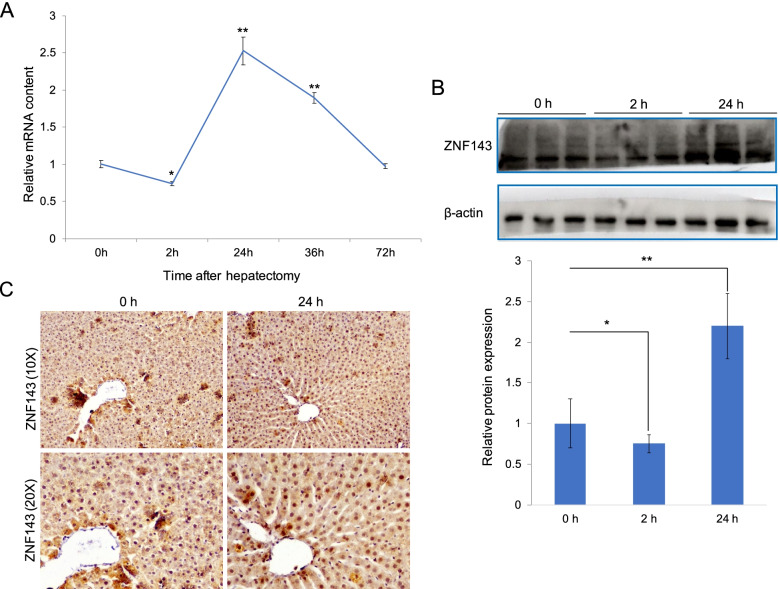
The expression of ZNF143 is upregulated during the proliferation phase of liver regeneration. **A** mRNA microarray data showing the relative expression level of ZNF143 during liver regeneration (*n* = 3 per time point in each group). **B** Western blot analyses showing ZNF143 protein level at 0 h, 2 h and 24 h during liver regeneration. Three biological replicates were analyzed. **C** IHC analyses showing the expression of ZNF143 in liver tissue during the proliferation phase of liver regeneration. The data are presented as mean ± SD. P values: *P < 0.05; **P < 0.01 using two-tailed Student t test (**A**, **B**)

To investigate the role of ZNF143 in liver cell proliferation, we previously detected ZNF143 mRNA expression in regenerating liver and regenerated hepatocytes by rat genome 230 2.0 microarray. Interestingly, ZNF143 expression in regenerating liver exhibited a dynamic alteration in response to 2/3 PH (Fig. 1A). It was downregulated during the priming phase (2 h) and then upregulated during the proliferation phase (24–72 h). The expression of ZNF143 mRNA in regenerated hepatocytes was almost same as that in regenerating liver (Fig. S1). The expression and localization of ZNF143, during the proliferation phase, were further determined using western blotting and IHC assays. Consistent with the microarray results, ZNF143 was upregulated and was widely expressed in liver tissues (Fig. 1B, C). Taken together, these data indicate that ZNF143 levels were upregulated during the proliferation phase of liver regeneration.

### ZNF143 OE inhibition of BRL-3A cell proliferation and cell cycle progression

**Fig. 2:**
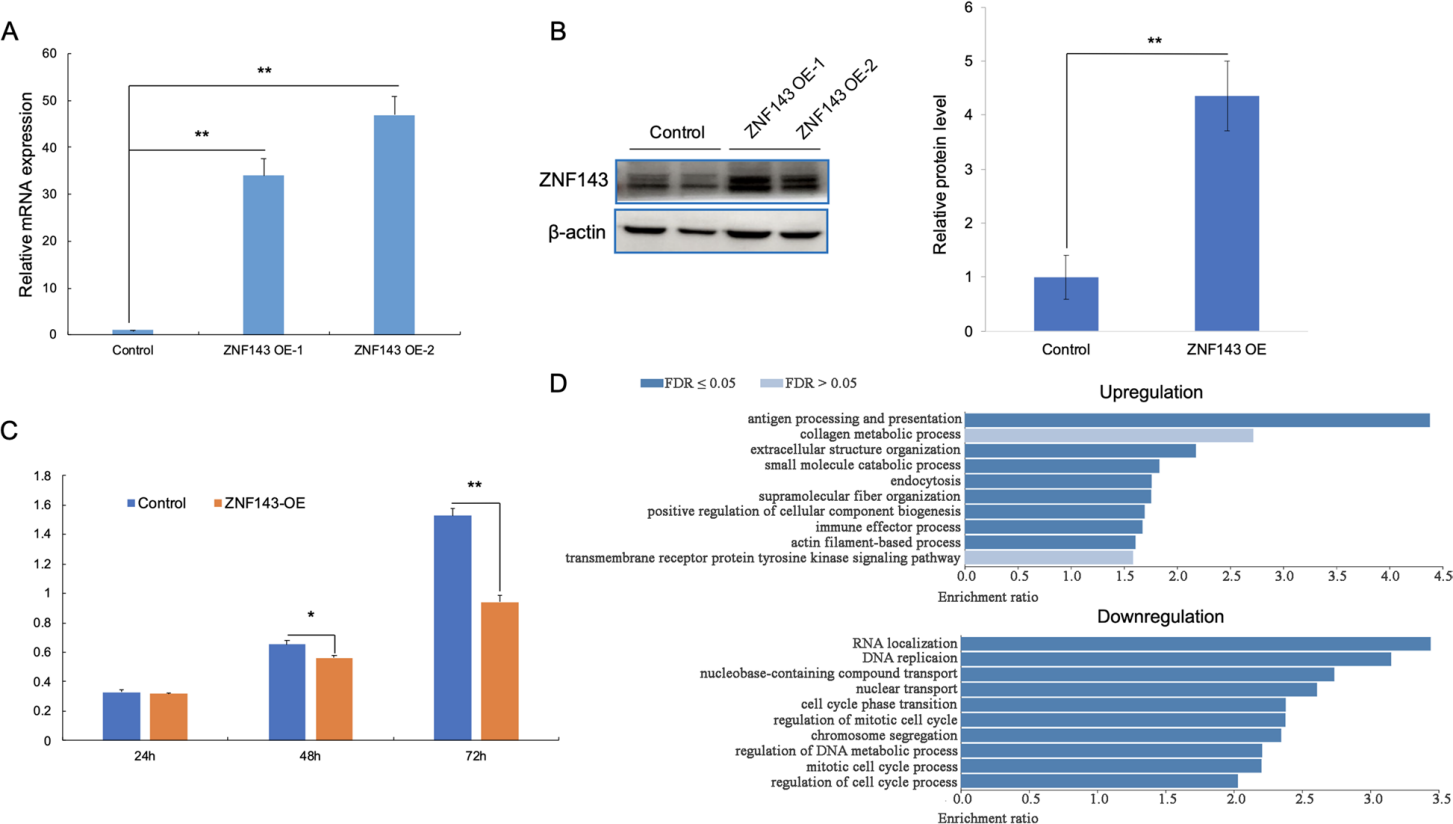
ZNF143 OE significant inhibition of BRL-3A cell proliferation and cell cycle progression. **A** qRT-PCR analyses showing ZNF143 mRNA level after OE For each qRT-PCR experiment, three biological replicates and three technical replicates were analyzed. β-actin was used as an internal reference gene. **B** Western blot analyses showing ZNF143 protein level after OE. Two biological replicates and three technical replicates were analyzed. **C** CCK-8 assay analyses showing cell proliferation 24 h, 48 h, and 72 h after ZNF143 OE. For each CCK-8 assay, three biological replicates were analyzed. **D** GO (RNA-seq) enrichment analyses showing cell biological pathways enriched 72 h after OE. The data are presented as mean ± SD. P values: *P < 0.05; **P < 0.01 using two-tailed Student t test (**A**, **B**, **C**); FDR ≤ 0.05 (**D**)

A stable ZNF143-overexpressing cell line (BRL-3A) was established to evaluate the biological role of ZNF143 during liver regeneration. Next, we determined the levels of ZNF143 mRNA and protein expression in ZNF143-overexpressing cells using qRT-PCR and western blotting. As expected, ZNF143 expression was significantly up-regulated (Fig. 2A, B). Cell proliferation after 24, 48, and 72 h was evaluated using CCK-8 assay and found that the proliferation of BRL-3A cells was significantly inhibited by ectopic expression of ZNF143, especially after 72 h (Fig. 2C). Cell proliferation was also measured by EdU incorporation.The result showed that ZNF143 OE significantly inhibited cell proliferation (Fig. S2). Next, transcriptome sequencing (RNA-seq) was performed to determine the effect of ZNF143 on cell proliferation. Two independent biological replicates, after 48 and 72 h, were carried out to investigate the effect of ZNF143 OE on cell proliferation. Correlation analysis indicated that all biological replicates of each sample were clustered together (Fig. S3). GO analysis of the data obtained from the 72 h RNA-seq revealed that all significantly downregulated genes were enriched in DNA replication- and cell cycle-related biological processes, suggesting that ZNF143 OE significantly inhibited BRL-3A cell proliferation and cell cycle progression (Fig. 2D and Table S1). However, genes related to immune response were mostly up-regulated (Fig. 2D). KEGG analysis showed that cell cycle-related pathways were enriched among down-regulated genes, whereas HCC-related signaling pathways and virus infection pathways were enriched among up-regulated genes (Fig. S4). Altogether, these findings suggested that ZNF143 OE inhibited normal liver cell proliferation.

### Genome-wide BS distribution analysis of ZNF143

**Fig. 3:**
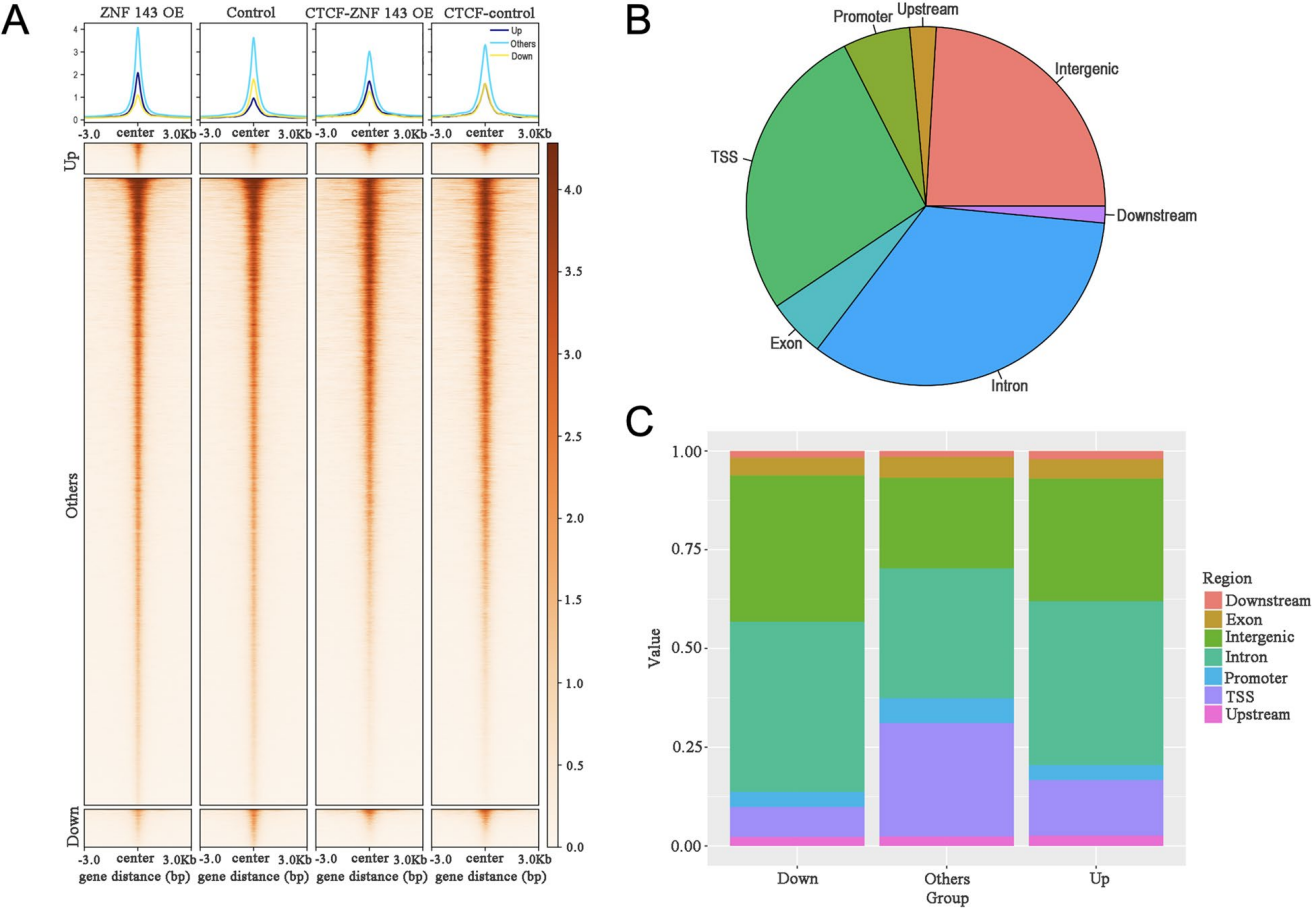
Genome-wide BS distribution analysis of ZNF143 after OE. **A** Heatmap showing the changes of ZNF143 and CTCF BSs after ZNF143 OE (FDR < 0.1, log_2_FoldChange > 0.5). **B** Genome-wide peak annotation showing the proportion of exon, intron, promoter, TSS and intergenic region after ZNF143 OE. **C** Differential peak annotation showing the proportion of exon, intron, promoter, TSS and intergenic region after ZNF143 OE

CUT&Tag assay was performed to explore the molecular mechanism of ZNF143 OE-mediated inhibition of liver cell proliferation. Correlation analysis of ZNF143 CUT&Tag data indicated that all biological replicates of each sample were clustered together (Fig. S5). Using peak calling statistical analyses, we obtained 22,580 ZNF143-BSs (Table S2). Next, ZNF143 occupancy in chromatin after ZNF143 OE was investigated. The BSs were divided into three groups based on the following criteria: (1) significantly enhanced after OE (Up), (2) significantly weakened after OE (Down), and (3) unchanged after OE (Others). Overall, approximately 10% of BSs significantly changed in ZNF143 CUT&Tag datasets (Fig. 3A and Table S2). Previous studies have indicated that ZNF143 is a key TF in the CTCF-bound promoter-enhancer loops. Therefore, it was important to investigate if ZNF143-OE affected the genomic binding of CTCF. Our CTCF CUT&Tag data analysis indicated that although most ZNF143 peaks overlapped with those of CTCF, its binding did not change significantly (Fig. 3A).

Based on data obtained from ZNF143 CUT&Tag, we demonstrated that most of the ZNF143-BSs were localized in the intron/intergenic regions (Fig. 3B). In addition, a considerable number of ZNF143 peaks were close to the transcription start sites (TSSs), suggesting that ZNF143 regulated gene expression mainly by binding to specific BSs in gene promoters (Fig. 3B and Table S2). Finally, we found that most of differential peaks (“Up” and “Down”) were distributed in the intron/intergenic region, and less in the TSS and promoter (Fig. 3C). In other words, although ZNF143 regulates gene expression mainly through direct binding to target promoters, it is more likely to be involved in long-range chromatin interactions after OE.

### Genes affected by ZNF143 differential BSs and their localization

**Fig. 4:**
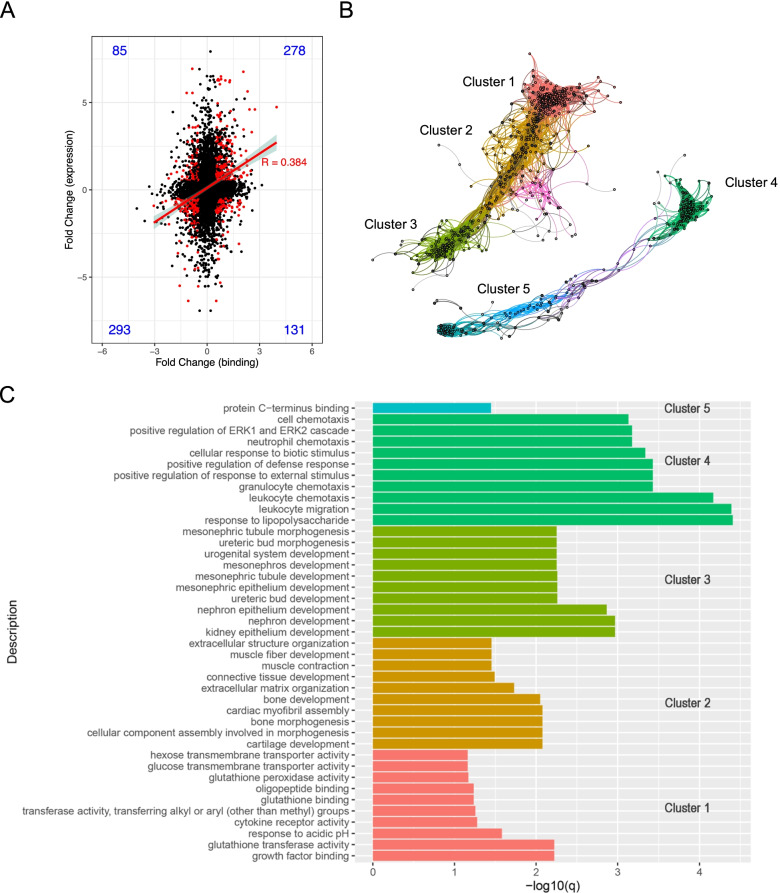
DEGs regulation analysis using integrated ChIP-seq and RNA-seq data. **A** The correlation between ZNF143 differential BSs and DEGs after ZNF143 OE. The red dot represents genes with significant differential expression (FDR < 0.05, log_2_FoldChange > 0.5) and significant differential binding (log_2_FoldChange > 0.5). Numbers within plots denote the percentage of genes in the respective quadrants. **B** Correlation network analysis of the DEGs (FDR < 0.01, log_2_FoldChange > 1.0). The black dot represents genes with significant differential binding (log_2_FoldChange > 0.5). **C** GO enrichment analysis of genes in (**B**)

Integrated RNA-seq and CUT&Tag data were utilized to demonstrate the relationship between ZNF143 binding and its effect on gene expression. There was a correlation between ZNF143 with significant differential binding strength (log_2_FoldChange > 0.5), and genes with significant differential expression (FDR < 0.05, log_2_FoldChange > 0.5) after ZNF143 OE (Table S3) (Fig. 4A, red dot, *R* = 0.384). The effect of differential BSs on the role and function of these differentially expressed genes (DEGs) was also investigated. Thus, a differentially expressed gene network was constructed and analyses of the genes of interest were performed. Interestingly, genes (black dot) (FDR < 0.01, log_2_FoldChange > 1) affected by differential BSs (log_2_FoldChange > 0.5) were located at the center of each gene expression module (Fig. 4B and Table S4). This also indicated that these genes were critical for gene expression network formation. Subsequently, GO enrichment and KEGG pathway analyses of the five gene clusters were performed. Among these, clusters 1, 2, and 3 belonged to the upregulated genes, and clusters 4 and 5 belonged to the downregulated genes (Table S3). Genes related to growth factor binding, extracellular structure/matrix organization, tissue and cell development, HCC, Wnt signaling pathway, and Hippo signaling pathway were enriched in clusters 1, 2, and 3, whereas genes related to positive regulation of defense response, cell chemotaxis, and IL-17, chemokine, and TNF signaling pathways were enriched in clusters 4 and 5 (Fig. 4C and Fig. S6). Furthermore, pathways enriched in clusters 1, 2, and 3 were important for the maintenance of liver cell identity. Collectively, ZNF143 OE might have caused a partial reorganization of the gene expression modules. However, after ZNF143 OE, results showed that the DEGs were affected by differential binding regulation of ZNF143 and were not directly related to cell proliferation inhibition.

## Discussion

Previous studies have shown that ZNF143 expression is associated with cell cycle regulation, cell proliferation, cancer development, and DNA repair. However, the role of ZNF143 in liver regeneration and normal liver cells remains unclear. In this study, ZNF143 expression was significantly upregulated during the proliferation phase of liver regeneration. We demonstrated that ZNF143 OE in normal rat liver cells (BRL-3A) inhibited cell proliferation. The BRL-3A cell line is commonly used to study the function of rat liver regeneration and hepatocytes in vitro [34]. Meanwhile, we found that the effect of ZNF143 on proliferation varied at different time points during in vitro culture of stable BRL-3A cell lines overexpressing ZNF143. As a TF, the function of ZNF143 is tightly linked to its ability to detect its respective BS in the genome. Thus, our results suggest that genes affected by ZNF143 differential BSs at different time points are partial differences. In addition, it may be related to the delayed effects of ZNF143 protein expression. In particular, genes related to cell proliferation and cell cycle were significantly affected at 72 h than at 48 h. Integrative analysis of RNA-seq and CUT&Tag data resulted in a correlation between ZNF143 binding and effects on gene expression, and genes affected by ZNF143 differential BSs were located in the center of each gene expression module. Further analysis indicated that ZNF143 OE may have an important effect on some of the most important signaling pathways that involved in liver regeneration and cancer development, such as the Wnt and Hippo signaling pathways.

A recent study showed that ZNF143 is overexpressed in HCC tissues and promotes HCC cell proliferation and cell cycle transition [27]. Our study indicated that the HCC-related signaling pathways were significantly enriched by OE of ZNF143 in normal rat liver cells (Fig. S6). This high expression suggests that ZNF143 promotes the formation of hepatoma cells. From these results, we can speculate that ZNF143 OE in normal liver cells can inhibit cell proliferation, whereas its high expression in hepatoma cells can promote cell proliferation. Taken together, we concluded that the expression level of ZNF143 in the liver is important for the maintenance of cell identity.

CTCF is a key higher-order chromatin organizer. ZNF143 colocalizes with CTCF and helps establish CTCF loops [24, 26]. The current study investigated the effects of ZNF143 OE on CTCF binding to chromatin. The results showed that CTCF binding did not change significantly after ZNF143 OE. This indicates that CTCF binding to DNA is not easily affected by external conditions and is critical for maintaining normal gene expression. Moreover, the ZNF143 CUT&Tag datasets showed that the genome-wide differential BS signal of ZNF143 was not very strong after ZNF143 OE. This indirectly suggests that ZNF143 OE does not only affect its binding to chromatin. Interestingly, confocal IF staining and nucleocytoplasmic separation assay showed that ZNF143 signaling in the cytoplasm was enhanced by ZNF143 OE (Fig. S7A, V). Thus, it is likely that OE of ZNF143 enhances its RNA-binding ability, thereby indirectly regulating gene expression and cell proliferation. However, this intriguing observation requires further investigation.

In conclusion, ZNF143 is a ubiquitously expressed TF that regulates a wide range of biological functions, including its role as a potential mitotic bookmarker during the cell cycle as shown in our recent report [35]. Although the current study found that ZNF143 OE inhibited normal liver cell proliferation, its role and molecular mechanisms in liver regeneration require further investigation.

## Supplementary Information

**Additional file 1:Fig. S1.** Therelative expression level of ZNF143 in hepatocytes during liver regeneration (n=3 per time point in each group). The data are presented as mean ± SD. P values:*P < 0.05; **P < 0.01 using two-tailed Student t test.

**Additional file 2:Fig. S2.** EdU incorporation was assessed by immunofluorescence. Quantification ofEdU incorporation (Red) reveals a significant decrease in EdU incorporation.Data presented as percent cells with EdU staining and include three biologicalreplicates. Thedata are presented as mean ± SD. P values: *P < 0.05 using two-tailedStudent t test.

**Additional file 3:Fig. S3.** Correlation analysis of RNA-seq data (including 48h and 72h).

**Additional file 4: Fig.S4.** KEGG pathway analysis of RNA-seq data (72h).

**Additional file 5:Fig. S5.** Correlation analysis of CUT&Tag data (including 48h and 72h).

**Additional file 6: Fig.S6.** KEGG pathway analysis of genes in Fig. 4B.

**Additional file 7: Fig.S7.** Distribution of ZNF143 expression. (A) Confocal microscopy analysis of ZNF143intracellular distribution after OE. The chromatin was stained using DAPI (blue), and anti-ZNF143was stained using Cy-3 (red). (B) Nucleocytoplasmic separation of ZNF143 innormal control BRL-3A cells and stable BRL-3A cell lines overexpressing ZNF143.Lamin A/C and GAPDH were used as internal control for nucleocytoplasmicseparation.

**Additional file 8: TableS1.** A list of DEGs at 72h.

**Additional file 9: TableS2.** All peaks of ZNF143.

**Additional file 10: TableS3.** A list of all DEGs at 48 h and 72 h.

**Additional file 11: TableS4.** A list of net_node_gene from Fig. 4A.

## Data Availability

All sequencing data (RNA-seq and CUT&Tag) generated in this study have been submitted to NCBI with a BioProject accession number of PRJNA824936.

## Abbreviations

TF: Transcription factor
ZNF143: Zinc finger protein 143
CTCF: CCCTC-binding factor
OE: Overexpression
BS: Binding site
DEGs: Differentially expressed genes
HCC: Hepatocellular cancer
NAFLD: Nonalcoholic fatty liver disease
ALD: Alcohol-related liver disease
IF: Immunofluorescence
IHC: Immunohistochemistry
