# Supplementary figures and images for: The role of ZNF143 overexpression in rat liver cell proliferation
Source: BMC Genomics. 2022 Jul 2;23:483. doi: 10.1186/s12864-022-08714-2 (PMC9250731; doi:10.1186/s12864-022-08714-2)

Relative mRNA content

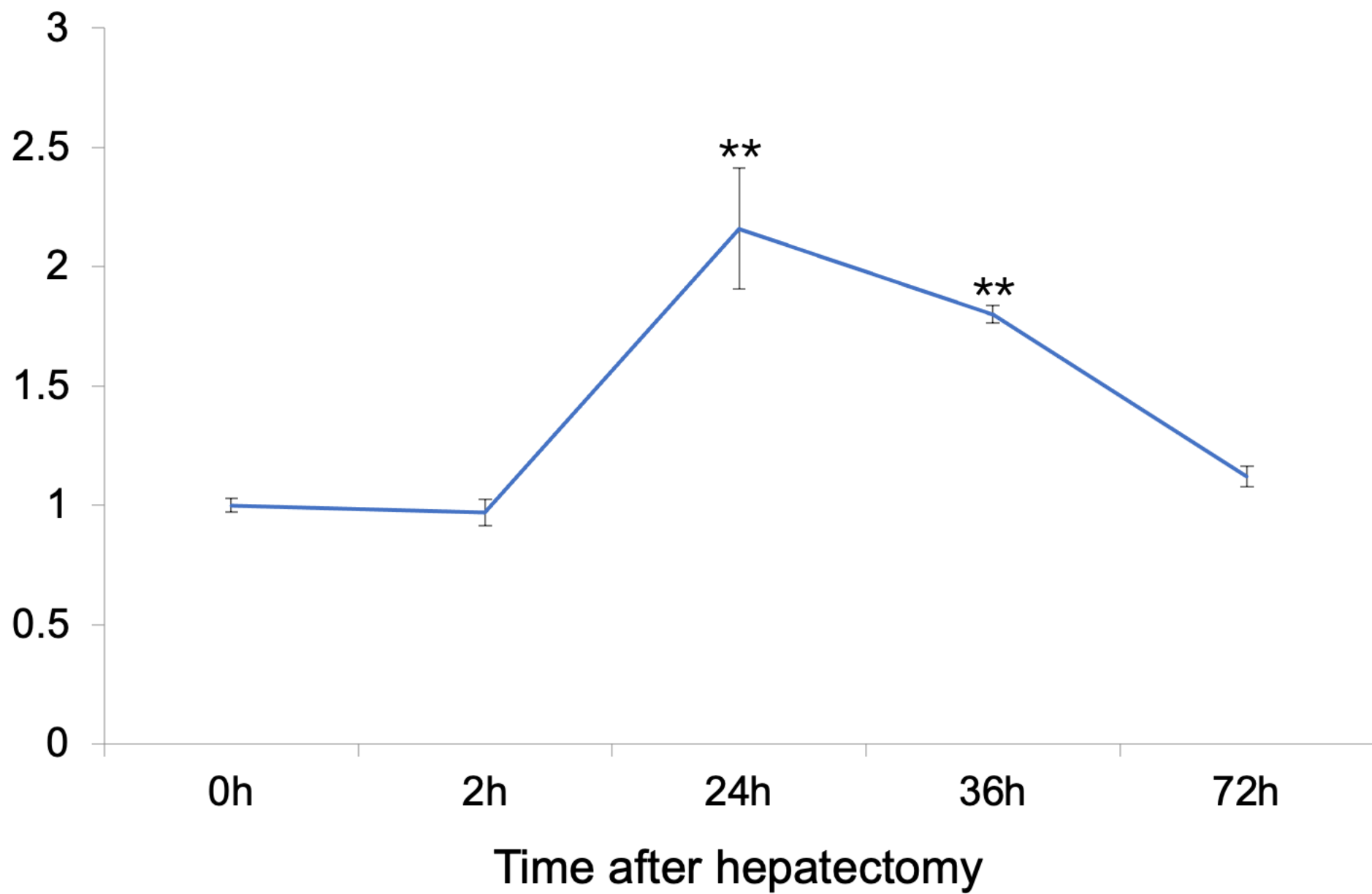

Supplement: Supplementary file 1 — Additional file 1:Fig. S1. Therelative expression level of ZNF143 in hepatocytes during liver regeneration (n=3 per time point in each group). The data are presented as mean ± SD. P values:*P < 0.05; **P < 0.01 using two-tailed Student t test. [file 12864_2022_8714_MOESM1_ESM.pdf]

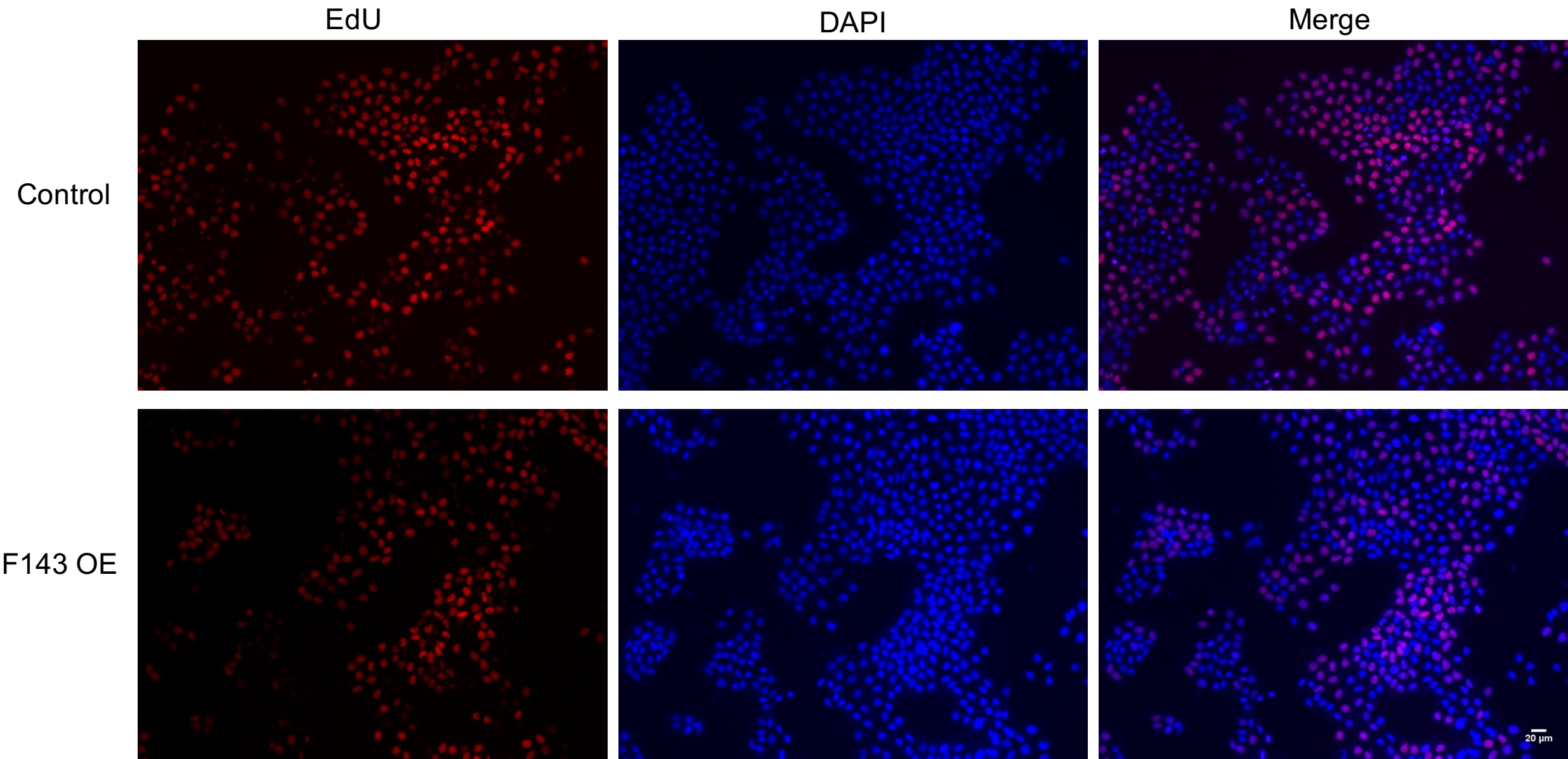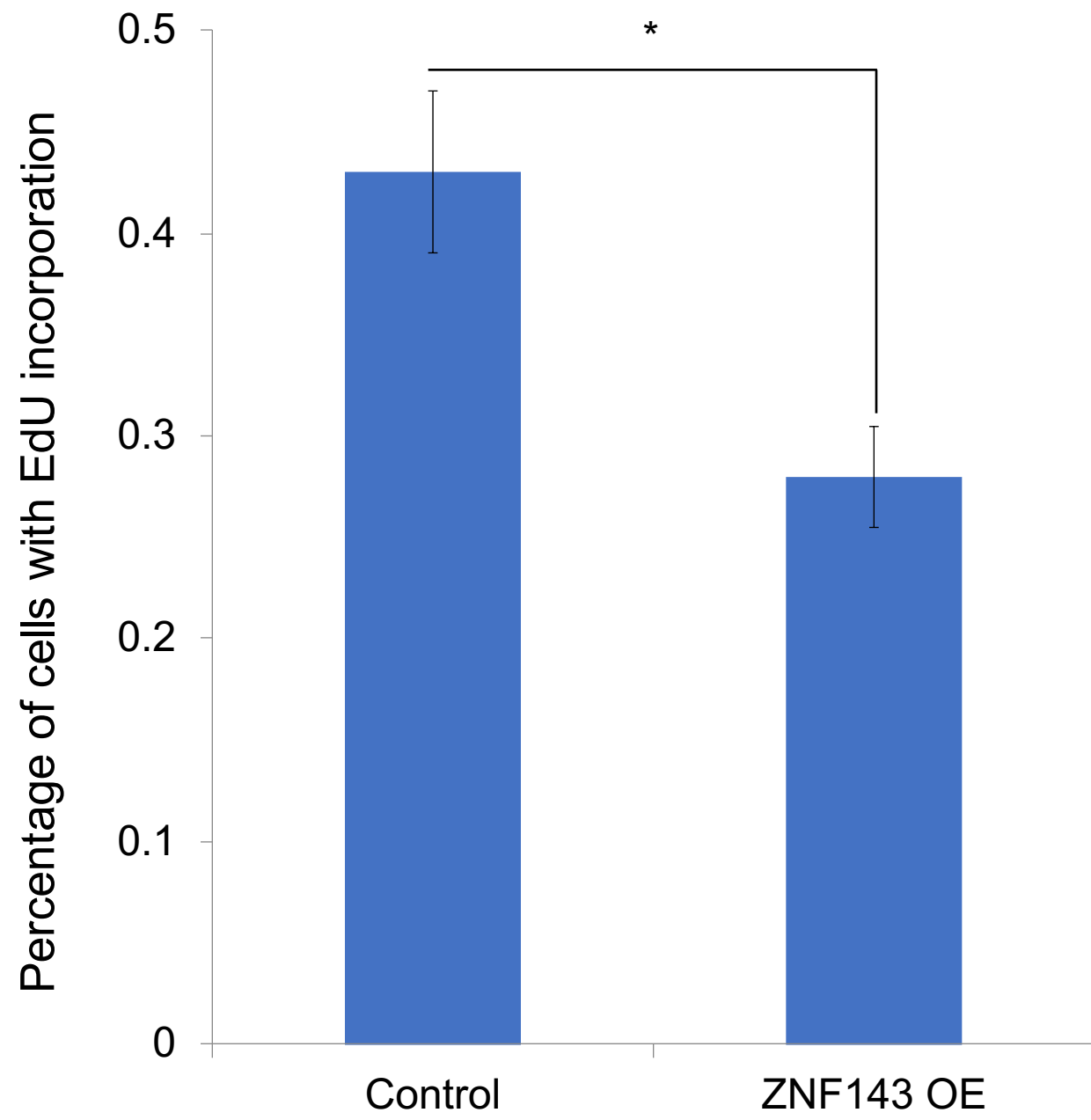

Supplement: Supplementary file 2 — Additional file 2:Fig. S2. EdU incorporation was assessed by immunofluorescence. Quantification ofEdU incorporation (Red) reveals a significant decrease in EdU incorporation.Data presented as percent cells with EdU staining and include three biologicalreplicates. Thedata are presented as mean ± SD. P values: *P < 0.05 using two-tailedStudent t test. [file 12864_2022_8714_MOESM2_ESM.pdf]

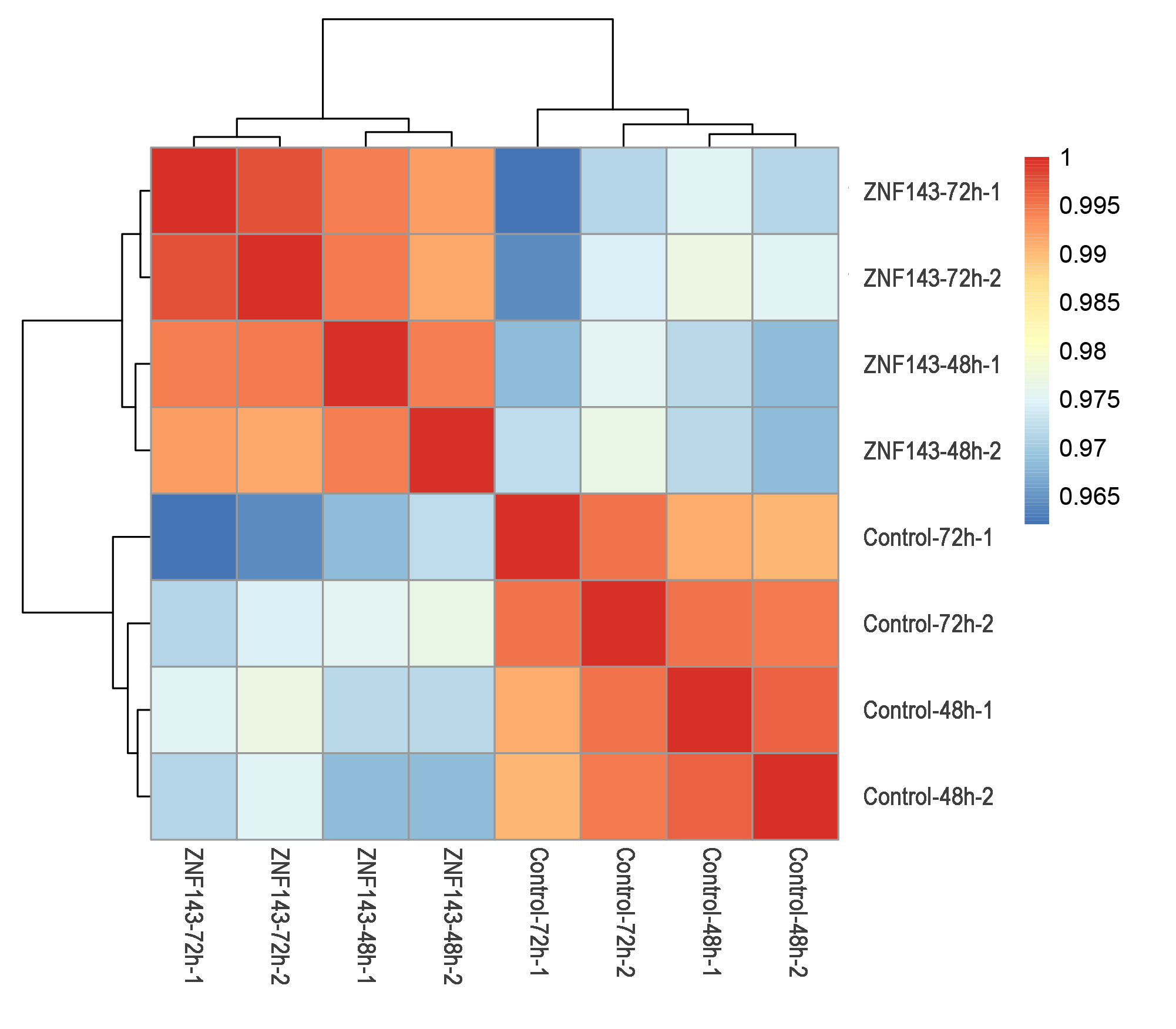

Supplement: Supplementary file 3 — Additional file 3:Fig. S3. Correlation analysis of RNA-seq data (including 48h and 72h). [file 12864_2022_8714_MOESM3_ESM.png]

FDR  $\leq$  0.05    FDR > 0.05

## Upregulation

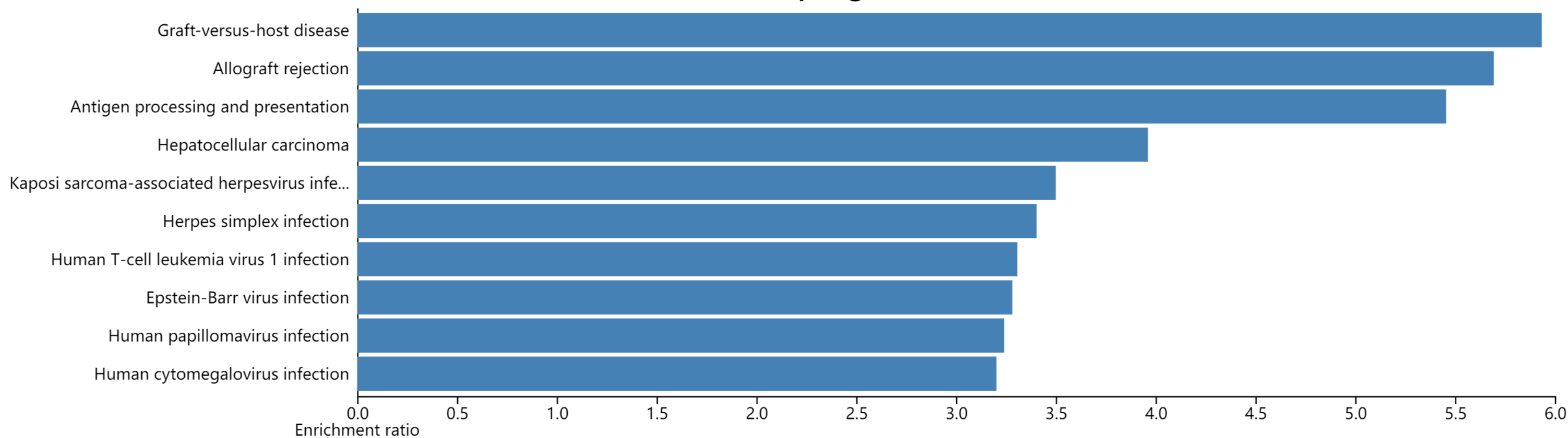

## Downregulation

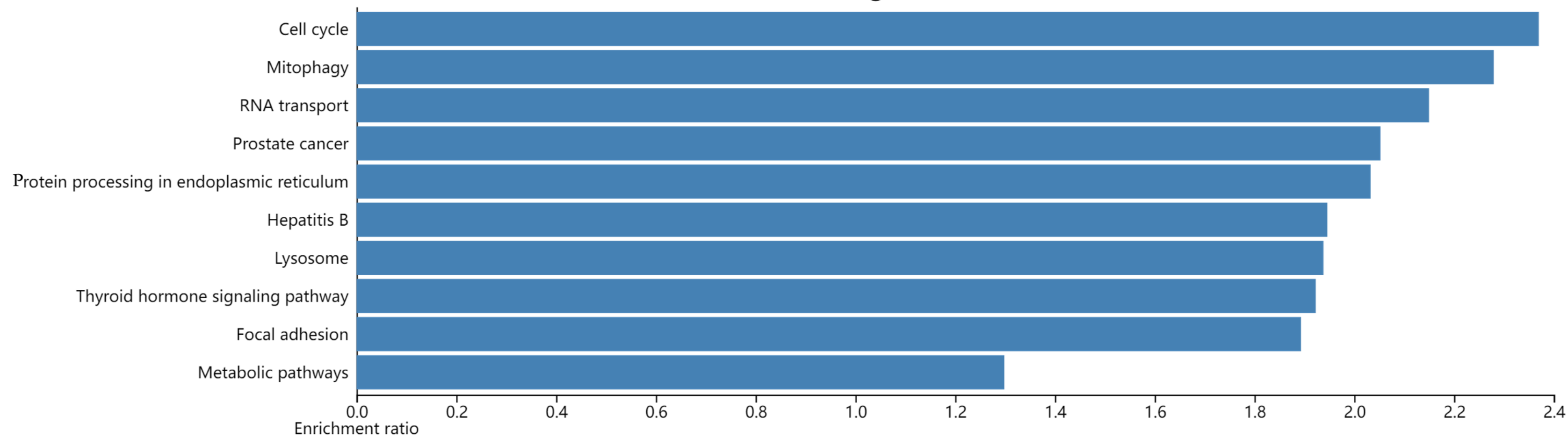

Supplement: Supplementary file 4 — Additional file 4: Fig.S4. KEGG pathway analysis of RNA-seq data (72h). [file 12864_2022_8714_MOESM4_ESM.pdf]

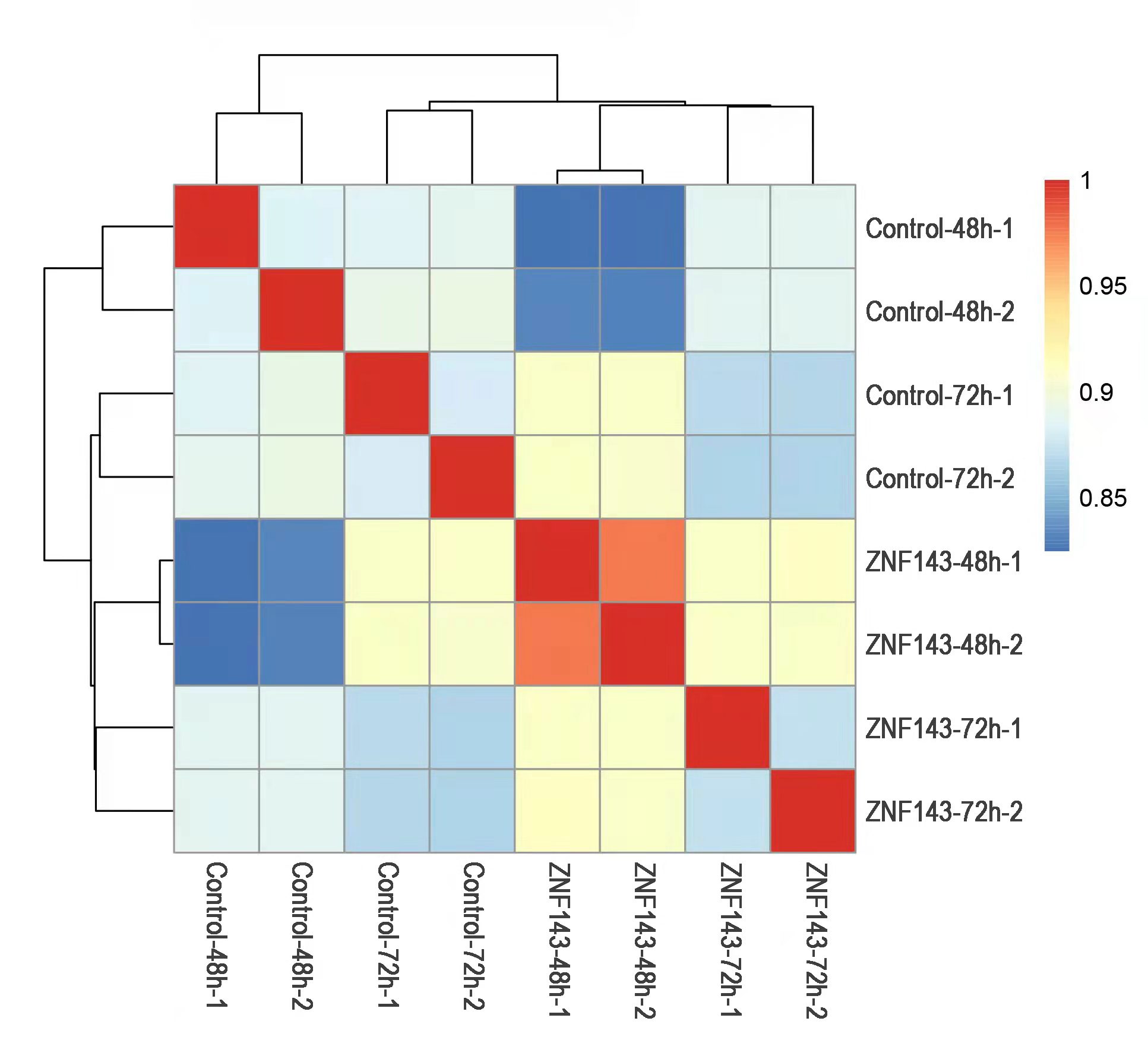

Supplement: Supplementary file 5 — Additional file 5:Fig. S5. Correlation analysis of CUT&Tag data (including 48h and 72h). [file 12864_2022_8714_MOESM5_ESM.png]

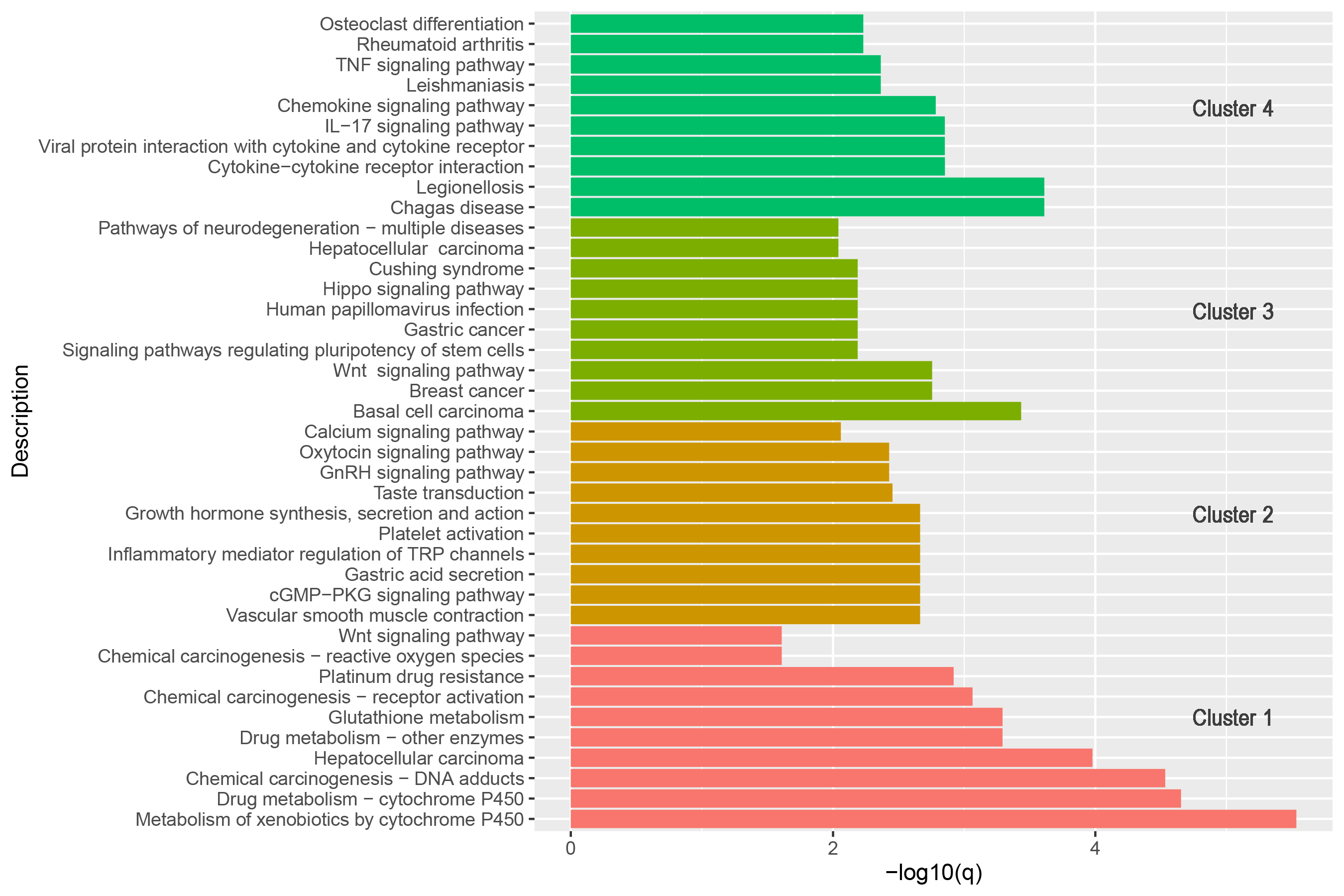

Supplement: Supplementary file 6 — Additional file 6: Fig.S6. KEGG pathway analysis of genes in Fig. 4B. [file 12864_2022_8714_MOESM6_ESM.png]

A

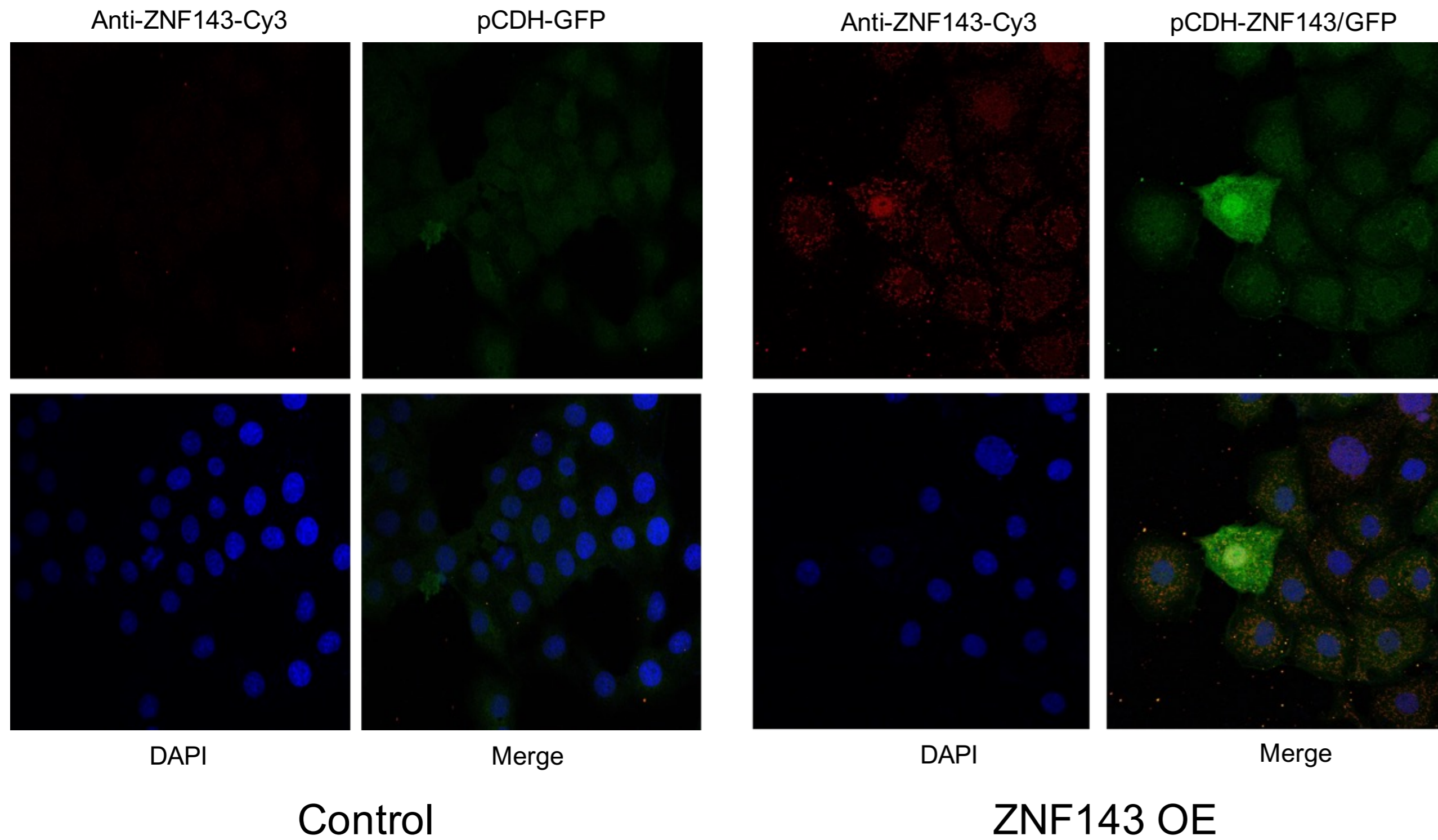

B

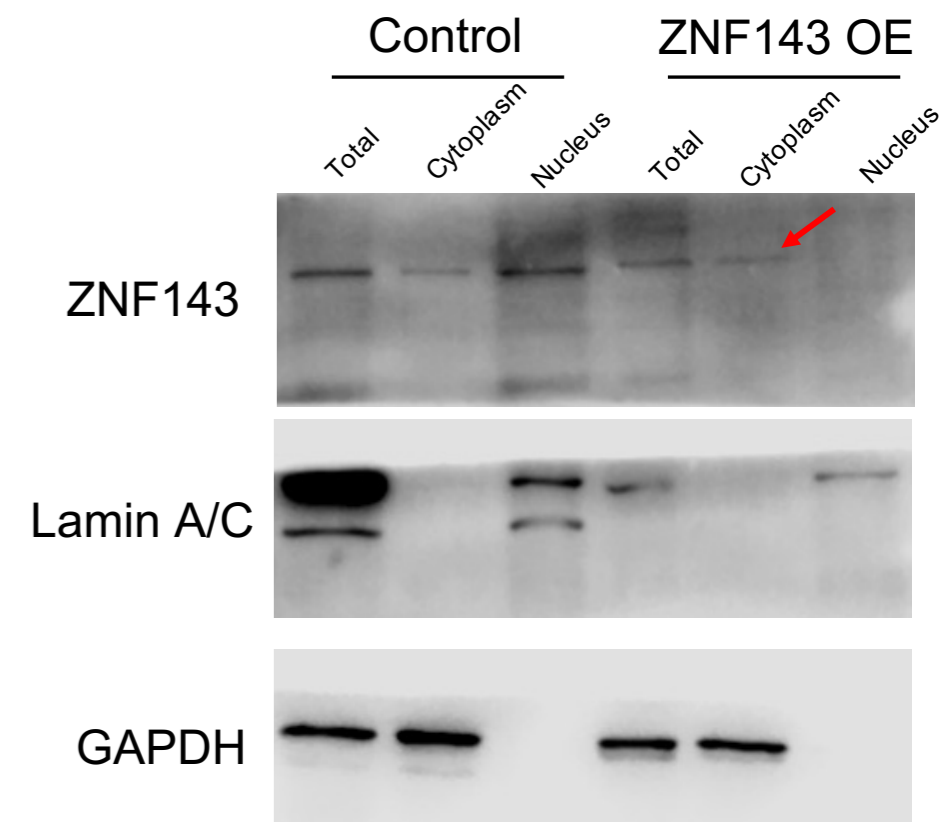

Supplement: Supplementary file 7 — Additional file 7: Fig.S7. Distribution of ZNF143 expression. (A) Confocal microscopy analysis of ZNF143intracellular distribution after OE. The chromatin was stained using DAPI (blue), and anti-ZNF143was stained using Cy-3 (red). (B) Nucleocytoplasmic separation of ZNF143 innormal control BRL-3A cells and stable BRL-3A cell lines overexpressing ZNF143.Lamin A/C and GAPDH were used as internal control for nucleocytoplasmicseparation. [file 12864_2022_8714_MOESM7_ESM.pdf]
